# The Principles and Practice of Ophthalmic Medicine and Surgery

**Published:** 1847-04

**Authors:** 


Art. VII.—*The Principles and Practice of Ophthalmic Medicine and Surgery.* By T. Wharton Jones, F.R.S., Lecturer on Anatomy, Physiology and Pathology at the Charing Cross Hospital; Foreign Member of the Royal Medical Society of Copenhagen; Corresponding Member of the Imperial Royal Medical Society of Vienna, etc. etc. With one hundred and two Illustrations. *Edited by* Isaac Hays, M.D., Surgeon to Wills Hospital, etc. pp. 507, 12mo. Philadelphia. Lea and Blanchard. 1847.

This is one of a series of manuals which has attained great popularity in our country. The series embraces Ferguson's Surgery, Carpenter's Physiology, Wilson's Anatomy, Taylor's

Medical Jurisprudence, Fownes's Chemistry, Royle's Materia Medica and Therapeutics, and Golding Bird's Nat. Philosophy. All these works have been noticed in this Journal except the last, and the one before us on Ophthalmic Medicine and Surgery. The design of these manuals is to afford the student a complete and comprehensive digest of the several subjects of which they treat in a convenient compass, and it is pretty conclusive proof of the successful execution of the design, that the works have been so well received. The present volume is a worthy companion of those which have preceded it, and, both in the care with which the matter has been prepared by its author, and the style in which it is issued by the American publishers, will commend itself to the profession. A real service has been rendered to science by the publication of the series of which it is one. The study of medicine has been made easier and more pleasant by the efforts of their various authors.

The volume before us will be found to embrace a full account of all the maladies and accidents incident to the eye, and, brief as it is, to furnish something of the history of ophthalmic medicine. It commences with directions as to the mode of exploring the eyes in order to a diagnosis. The exploration is of two kinds, termed *subjective*, and *objective;* the first comprehending an inquiry into the patient's sensations in the affected organ, such as pain, tolerance of light, and state of vision; and the latter being directed towards the morbid conditions which admit of being perceived by the surgeon. These conditions, so far as they can be so represented, are illustrated by engravings. The application of remedies to the eyes, and the minor operations on these organs are then explained in detail, accompanying which are the formulas in best repute in ophthalmic inflammations. Inflammation of the eyes is treated of at great length, and all the forms of it are illustrated, and the claims of the numerous remedies discussed. The subject embraces scrofulous, arthritic and variolous inflammation. The several operations necessary in diseases and deformities of the eye are described with minuteness. In a word, the *manual* is a complete one, and will be found equally convenient as a text book for the student, and as a work of reference for the practitioner.

